# Genome-wide analysis of rice ClpB/HSP100, ClpC and ClpD genes

**DOI:** 10.1186/1471-2164-11-95

**Published:** 2010-02-08

**Authors:** Amanjot Singh, Upasana Singh, Dheeraj Mittal, Anil Grover

**Affiliations:** 1 Department of Plant Molecular Biology, University of Delhi South Campus, New Delhi-110021, India

## Abstract

**Background:**

ClpB-cyt/HSP100 protein acts as chaperone, mediating disaggregation of denatured proteins. Previous studies have shown that ClpB-cyt/HSP100 gene belongs to the group class I Clp ATPase proteins and ClpB-cyt/HSP100 transcript is regulated by heat stress and developmental cues.

**Results:**

Nine ORFs were noted to constitute rice class I Clp ATPases in the following manner: 3 ClpB proteins (ClpB-cyt, Os05g44340; ClpB-m, Os02g08490; ClpB-c, Os03g31300), 4 ClpC proteins (ClpC1, Os04g32560; ClpC2, Os12g12580; ClpC3, Os11g16590; ClpC4, Os11g16770) and 2 ClpD proteins (ClpD1, Os02g32520; ClpD2, Os04g33210). Using the respective signal sequences cloned upstream to GFP/CFP reporter proteins and transient expression studies with onion epidermal cells, evidence is provided that rice ClpB-m and Clp-c proteins are indeed localized to their respective cell locations mitochondria and chloroplasts, respectively. Associated with their diverse cell locations, domain structures of OsClpB-c, OsClpB-m and OsClpB-cyt proteins are noted to possess a high-level conservation. *OsClpB-cyt *transcript is shown to be enriched at milk and dough stages of seed development. While expression of *OsClpB-m *was significantly less as compared to its cytoplasmic and chloroplastic counterparts in different tissues, this transcript showed highest heat-induced expression amongst the 3 ClpB proteins. OsClpC1 and OsClpC2 are predicted to be chloroplast-localized as is the case with all known plant ClpC proteins. However, the fact that OsClpC3 protein appears mitochondrial/chloroplastic with equal probability and OsClpC4 a plasma membrane protein reflects functional diversity of this class. Different class I Clp ATPase transcripts were noted to be cross-induced by a host of different abiotic stress conditions. Complementation assays of *Δhsp104 *mutant yeast cells showed that *OsClpB-cyt*, *OsClpB-m*, *OsClpC1 *and *OsClpD1 *have significantly positive effects. Remarkably, *OsClpD1 *gene imparted appreciably high level tolerance to the mutant yeast cells.

**Conclusions:**

Rice class I Clp ATPase gene family is constituted of 9 members. Of these 9, only 3 belonging to ClpB group are heat stress regulated. Distribution of ClpB proteins to different cell organelles indicates that their functioning might be critical in different cell locations. From the complementation assays, OsClpD1 appears to be more effective than OsClpB-cyt protein in rescuing the thermosensitive defect of the yeast *ScΔhsp104 *mutant cells.

## Background

Heat stress threatens future prospects of increased grain production in crops. Rice (*Oryza sativa*) is most important world food crop. The production of rice is getting severely affected with increases in mean global temperature. According to estimates, yield of rice declines by 10% for every 1°C increase in growing period minimum temperature in the dry season [1]. Processes like spikelet fertility, grain quality and yield processes in rice are considered to be especially sensitive to heat stress [2]. For breeding heat tolerant rice, it is important that molecular components that underlie the heat shock response in this species are understood [3]. Microarray profiling data have shown that heat stress response in maturing tomato microspores involves heat shock proteins, ROS scavengers, hormones and sugars [4]. Understandably, the major molecular changes in rice plants as affected by heat stress need to be worked out. This is especially relevant since rice has emerged as a model plant species of the group monocots due to its small genome size, availability of large collection of full-length cDNAs (FL-cDNAs) and for the fact that the whole genome of this plant species is completely sequenced. This crop has attracted a great deal of efforts for the elucidation of gene functions; completion of genome sequencing in rice has paved way for comprehensive functional characterization of genes, transcription factors, signaling components and promoters [5,6]. The completed rice genome sequence has been used for the characterization of a large number of gene families involved in diverse processes and pathways. However, with almost 42000 genes [7], several of them unknown, there are ample proteins still left to be characterized in this important crop species. In recent years, comprehensive details on heat shock regulated rice HSP20, HSP70, HSP90 and HSF gene families have been reported [8-11]. HSP100 is a major heat-regulated protein family in diverse organisms. Across the living systems, common features of HSP100 chaperone action include transient interactions with non-native protein species, in the prevention of aggregation and promotion of correct folding and assembly, or in unfolding for translocation or targeting to proteases. Singla and Grover [12] showed that homologues of yeast HSP104 protein are expressed in heat shocked rice seedlings. It was subsequently established that apart from heat, rice HSP100 expression is developmentally-controlled as seeds and developing embryos of rice show high constitutive levels of this protein [13,14].

HSP100 proteins belong to ClpB family [15-17]. Clp ATPases maintain quality of cellular proteins by performing the function of molecular chaperones and energy dependent proteases. Clp (**C**aseino**l**ytic **P**rotease) system was first identified as a heat shock inducible, multicomponent, ATP-dependent protease complex able to hydrolyze casein [18,19]. Subsequent studies showed that the Clp system can hydrolyze numerous other proteins and peptides in both aggregated and non-aggregated forms [20] Clp ATPases fall within the AAA^+ ^superfamily of ATPases associated with a substantially broader range of biological processes [21,22]. Class I ATPases (ClpA, ClpB, ClpC, ClpD) have two ATP binding domains and class II Clp ATPases (ClpM, ClpN, ClpX, ClpY) have one ATP binding domain [23]. Clp proteins are localized in various cellular organelles in plants [24]. Basically, Clp system members include three non-homologous gene families: ClpABCXY, ClpP and ClpQ. ClpACX members (but not ClpB) facilitate the activity of ClpP and some, such as ClpA [25] and ClpX [26], can function as independent chaperones in roles analogous to those of DnaK and DnaJ proteins. In contrast, ClpP proteolytic subunit exhibits low levels of peptidolytic activity. Further, when ClpP is complexed with ClpA, ClpC or ClpX, active holoenzymes which are able to cleave denatured proteins are formed. ClpAP, ClpXP and HslUV proteases are similar in design to the eukaryotic 26 S proteasome, with the ATPase subunits guarding the entrance to the proteolytic chamber [27,28]. Clp proteases in bacterial and eukaryotic systems have been implicated in vital cellular processes such as sporulation, DNA replication, protein turnover, stress tolerance and acclimation and regulation of gene expression [29]. ClpB is different from ClpA, ClpX and HslU as it does not associate with peptidase subunits. The function of ClpB is also distinct from that of other Clp ATPases: this protein is not involved in protein degradation [30], instead it disaggregates and reactivates strongly aggregated proteins [31-33]. The aggregation reversing activity of ClpB requires cooperation with the HSP70/HSP110 chaperone machinery [34].

In *Arabidopsis*, ClpB proteins have been divided into 3 classes according to cytoplasmic, chloroplastic and mitochondrial isoforms. Cytoplasmic ClpB is constituted by At1g74310, chloroplastic ClpB by At5g15450 and mitochondrial ClpB by At2g25140 [17]. Much of the work on ClpB proteins has been carried out on AtClpB-cyt isoform [35-38]. We isolated and sequenced rice ClpB-cyt gene (GenBank accession no. AJ316025) and showed that rice ClpB-cyt cDNA complements yeast *hsp104 *deletion and loss of thermotolerance trait in yeast [39]. Genetic analysis has shown that ClpB-cyt protein is essential for heat tolerance in plants. Mutants of *Zea mays *and *A. thaliana *plants under-expressing their respective ClpB-cyt/HSP100 proteins are observed to lack both basal as well as induced thermotolerance [35-37]. Queitsch et al [38] produced transgenic *A. thaliana *plants by modifying level of AtClpB-cyt/HSP100 protein. Transgenic plants in the latter study survived as high as 45°C (1 h) temperature stress as they showed vigorous growth after the removal of stress. Katiyar-Agarwal et al [40] over-expressed AtClpB-cyt/HSP100 protein in rice plants. The transgenic rice lines showed re-growth in the post-high temperature stress recovery phase while the untransformed plants could not recover to the similar extents. Keeler et al [41] reported cloning of a chloroplastic *HSP100/ClpB *gene from *Phaseolus lunatus*. The corresponding protein was seen to be localized to chloroplast. The accumulation of ClpB/HSP100 in this plant species was tightly correlated with heat acclimation, suggesting that the chloroplastic ClpB/HSP100 may be an important protein for acquiring thermotolerance. Chloroplastic ClpB from *Lycopersicon esculentum *has been shown to be important in acquired thermotolerance [42]. Antisense suppression of *LeHSP100/ClpB *in tomato plants made the plants more heat sensitive. Lee et al [17] noted that *Arabidopsis *knockout *ClpB-p *plants turn yellow on heat treatment. *ClpB-p *mutant plants showed a distinct change in chloroplast morphology upon subjecting plants to high temperature stress: chloroplasts were irregular to roundish in shape and were 21.5% of the size of normal chloroplasts. Lee et al [17] reported that At2g25140 (*ClpB-m*) was localized to mitochondria based on experiments in which transgenic *Arabidopsis *plants produced with signal peptide of At2g25140 fused with GFP showed expression of the reporter gene in mitochondria. This group further noted that the *ClpB-m *transcript was up-regulated during heat shock and the level of induced transcript for *ClpB-m *was lower as compared to *AtHSP100 *and *ClpB-p *transcripts. *ClpB-m *knockout *Arabidopsis *plants grew in comparable way to the wild type plants both during induced and acquired thermotolerance.

Plant ClpC proteins, which are the homolog of the *E. coli *ClpA proteins, have been noted in chloroplast stroma of several plant species [43-45]. ClpCs are considered to be highly conserved proteins among different species [46]. ClpC of *Bacillus subtilis *has been found not only to be involved in the removal of misfolded and aggregated proteins but also controls through regulated proteolysis, several key steps of development [47]. Two nearly-identical ClpC isomers exist in *Arabidopsis*. ClpC has been seen to be associated with ClpP in the stroma [48,49] in an ATP dependent manner [50]. Sjogren et al [51] reported that *clpC1 *mutant *Arabidopsis *plants display a retarded growth phenotype and leaves with a homogeneous chlorotic appearance. This mutant also exhibited fewer photosystem I and photosystem II complexes. ClpC in *Synechococcus elongatus *has been noted to possess ATPase activity as well as function as a molecular chaperone without the need of additional chaperones or adaptor proteins [52]. It is suggested that ClpC functions in part as a housekeeping chaperone *in vivo*, protecting unfolded, newly synthesized (or recently imported as in chloroplasts) polypeptides from aggregation. ClpD proteins differ from ClpCs by specific signature sequence and by its differential expression characteristics [23,44]. ClpD in *Arabidopsis *has previously been referred to as ERD1 [44,53,54] and SAG15 [55]. Weaver et al [56] reported that the ERD1 protein declines while its mRNA increases during senescence in *Arabidopsis*.

It is hence clear that plant Clps are associated with stress and developmental processes. The comprehensive analysis of rice Clp ATPases with respect to their structural and functional aspects has not been addressed to as yet. This study provides information on rice class I Clp ATPases with respect to genomic organization, regulation, protein architecture and cellular functions.

## Results

### Genome complexity of rice class I Clp ATPase members

The protein sequence of rice ClpB/HSP100 (accession number AJ316025) was noted to be same as the protein encoded by the MSU locus Os05g44340. Os05g44340 was used as a query for identifying the other Clp homologs at the MSU rice database. Nine ORFs encoded by Os02g08490, Os02g19450, Os02g32520, Os03g31300, Os04g32560, Os04g33210, Os11g16590, Os11g16770 and Os12g12850 loci were noted to share homology to Os05g44340 in this analysis (Table 1). Search using HSP100 as a key word showed that Os08g15230 is also one of the probable candidate genes belonging to Clp ATPase family. However, alignment of protein sequence of Os08g15230 with Os05g44340 showed that Os08g15230 does not contain most of the domains which are typically present in Clp proteins (except for the truncated ClpN domain). Therefore, Os08g15230 is not included as Clp ATPase family member herein. Further, Os02g19450 locus encoding for a mitochondrial 74kDa protein (HSP74) showed high similarity to yeast HSP78 protein. Though Os02g19450 protein appeared to be the closest homologue of Os05g44340 protein in terms of sequence similarity (59%), Os02g19450 lacked the N-terminal domain which other mitochondrial HSP78 proteins possess. Importantly, Os02g19450 locus appears not to be expressed in rice based on microarray as well as Quantitative-PCR (Q-PCR) analysis while the yeast HSP78 protein is strongly expressed in response to high temperature stress. We have therefore omitted Os02g19450 gene as well from the class I Clp ATPase family in further discussion. Overall then, we assume that 9 ORFs (i.e. Os02g08490, Os02g32520, Os03g31300, Os04g32560, Os04g33210, Os05g44340, Os11g16590, Os11g16770 and Os12g12850; Table 1) constitute rice class I Clp ATPases.

**Table 1:** Properties of rice class I Clp ATPase members. aa- amino acids; pI- isoelectric point.

Locus	Suggested nomenclature	Mol. Wt. (kDa), Length (aa), pI	Signal peptide (aa)	Predicted cellular localization	Gene length, ORF, introns
Os02g08490	ClpB-M	116.04, 1043, 7.26	87	Mitochondria	5072, 2952, 9
Os02g32520	ClpD1	101.8, 938, 7.02	83	Chloroplast	4465, 2817, 11
Os03g31300	ClpB-C	108.9, 978, 6.44	76	Chloroplast	6378, 2937, 14
Os04g32560	ClpC1	101.8, 918, 6.32	28	Chloroplast	5309, 2757, 8
Os04g33210	ClpD2	93.2, 858, 8.38	80	Chloroplast	5183, 2577, 9
Os05g44340	ClpB-Cyt	100.8, 912, 6.07	None	Cytoplasm/Nucleus	3106, 2739, 4
Os11g16590	ClpC3	100.8, 932, 8.24	48	Chloroplast/Mitochondria	5001, 2799, 8
Os11g16770	ClpC4	100.9, 918, 9.46	21	Plasma Membrane	5299, 2757, 8
Os12g12850	ClpC2	102.01, 919, 6.89	54	Mitochondria	4729, 2760, 8

### Phylogenetic relationship among plant class I Clp ATPases

**Figure 1:**
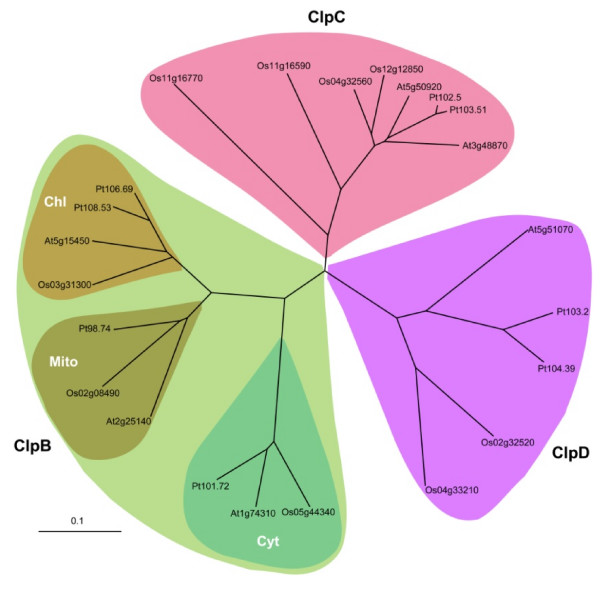
**Phylogenetic relatedness among different class I Clp ATPase proteins from rice, *Arabidopsis *and *Populus***. The phylogenetic tree was created using ClustalX 1.83, based on the predicted amino acid sequences. The branch lengths are proportional to divergence, with the scale of 0.1 representing 10% change.

Phylogenetic analysis of rice class I Clp ATPases with the corresponding *Arabidopsis *proteins (6 ORFs, details shown in Additional file 1) enabled us to annotate and classify the above 9 rice ORFs into ClpB, C and D classes as follows: 3 ClpB proteins (designated as B1, Os05g44340; B2, Os02g08490; B3, Os03g31300), 4 ClpC proteins (C1, Os04g32560; C2, Os12g12580; C3, Os11g16590; C4, Os1g16770) and 2 ClpD proteins (D1, Os02g32520; D2, Os04g33210). The extent of identity of these proteins with respect to Os05g44340 is as follows: Os02g08490-54.8%, Os02g32520-28.3%, Os03g31300-50.8%, Os04g32560-40%, Os04g33210-26.2%, Os11g16590-40.9%, Os11g16770-49.4% and Os12g12580-29.1%. *Populus trichocarpa *is the first tree species whose genome has been sequenced. A BLASTP search of *Populus *genome at http://genome.jgi-psf.org/Poptr1_1/ revealed that it has 8 homologs to rice Os05g44340. Clp ATPases from *Populus *have been named in this text as per the system described by Waters et al [57] for LMW HSPs. Molecular details for the 8 *Populus *Clp ATPases are shown in Additional file 1. Overall, we find that *Populus *genome contains 4 ClpB (Pt101.7, Pt106.7, Pt108.5 and Pt98.7), 2 ClpC (Pt102.5 and Pt103.5) and 2 ClpD (Pt103.2 and Pt104.4) genes. *Arabidopsis*-rice-*Populus *phylogenetic tree was constructed using amino acid sequences of 9 Clp ATPase proteins from rice (sequences downloaded from MSU rice database), 6 from *Arabidopsis *(sequences downloaded from MIPS database) and 8 from *Populus *(sequences downloaded from http://genome.jgi-psf.org/Poptr1_1/Poptr1_1.home.html using ClustalX and visualized using Treeview (Figure 1). The tree divided all 23 proteins into 3 major clades corresponding to ClpB, ClpC and ClpD proteins. ClpB clade was divided into 3 subclades according to cytoplasmic, chloroplastic and mitochondrial isoforms. Cytoplasmic ClpB are Os05g44340, At1g74310 and Pt101.72; chloroplastic ClpB are Os03g31300, At5g15450, Pt106.69 and Pt108.53 and mitochondrial ClpB are Os02g08490, At2g25140 and Pt98.74. At the amino acid level, Os02g08490 is 82.5% similar to At2g25140 which has recently been reclassified from AtClpB4 to AtClpB-M [17]. Os03g31300 showed high similarity (84%) with At5g15450. Os05g44340 has a similarity of 83.4% with At1g74310 at the amino acid level. ClpC clade contained 4 rice (Os04g32560, Os12g12850, Os11g16770 and Os11g16590), 2 *Arabidopsis *(At3g48870 and At5g50920) and 2 *Populus *(Pt102.5 and Pt103.51) members. At the amino acid level, Os04g32560 (OsClpC1) shows homology of 82.3 and 87.5% with At3g48870 and At5g50920, respectively. Os12g12850 (OsClpC2) shares 87.6% similarity with Os04g32560, the sequence being dissimilar only in the region towards N-terminus which corresponds to the signal peptide. Os12g12850 shows 83% and 86.5% identity at the amino acid levels with At3g48870 and At5g50920, respectively. Pt102.5 and Pt103.51 showed similarity in the range of 84-86% to Os04g32560 and Os12g12850, suggesting that ClpC ATPases are highly conserved across phyla. Os11g16770 and Os11g16590 also appear to be members of ClpC clade. ClpD class included two proteins from rice (Os02g32520 and Os04g33210), one from *Arabidopsis *(At5g51070) and two from *Populus *(Pt103.2 and Pt104.39). Os02g32520 has identity of 62.8% with At5g51070 which was previously identified as ERD1 protein and later rechristened as AtClpD [17]. Os04g33210 shows 60% sequence identity to At5g51070 at the amino acid level. Pt103.2 and Pt104.39 showed similarity ranging from 61-65% at the amino acid level with their rice counterparts. Table 1 provides detailed properties of rice class I Clp ATPase genes.

### Genomic organization of rice class I Clp ATPase genes

Rice class I Clp ATPase genes appear to be distributed in the genome (Table 1). There is no duplication event seen in these genes, suggesting that these genes have evolved independently from each other and there is no transposition involved. However, the evolutionary patterns of organellar and cytoplasmic isoforms of various class I Clp ATPases remain to be analyzed. Further, there is not much similarity among these genes at the level of distribution of exon and intron sequences: all class I Clp ATPase genes contained introns and there was no specific pattern in the arrangement of exons and introns in their genomic loci (Additional file 2). *OsClpB-c *has 15 exons and 14 introns which accounts for the most in any Clp member in rice class I ATPase gene family. *OsClpB-cyt *appears the smallest gene among rice class I Clp ATPase genes with respect to the genic region occupied. With respect to the proposed ORF of *OsClpC1 *gene, a discrepancy was noted: KOME database shows AK058510 to contain ORF of 1.107 kb while MSU database predicts ORF of 2.757 kb. While 2.757 kb ORF seems to encode the complete protein (918 amino acids; 101.8 kDa protein), there are no databank entries corresponding to 2.757 kb transcript of this gene. KOME clone in fact contains the last 1.211 kb region of the 2.757 kb clone. In this study, full length *OsClpC1 *was cloned from control unstressed rice seedling tissues using RT-PCR. Since a band corresponding to ~ 2.7 kb on 1% agarose gel in this reaction was noted (data not shown), it is thus indicated that *OsClpC1 *ORF is ~ 2.7 kb under the conditions examined herein.

### Domain structure of class I OsClp ATPase proteins

**Figure 2:**
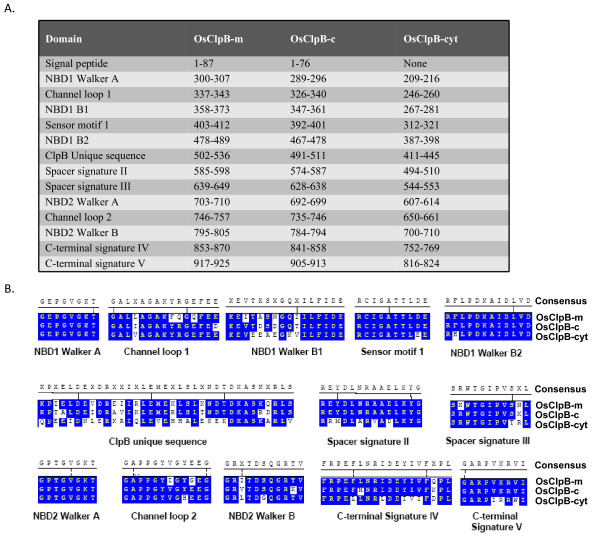
**A. Relative positions of different domains present in rice class I ClpB proteins**. The position of amino acids was marked manually after aligning the sequences in MegAlign module of DNASTAR. B. Consensus sequence of the different domains present in rice class I ClpB proteins. Alignments were done using ClustalV of MegAlign module of DNASTAR and alignment pictures were modified for representation.

**Figure 3:**
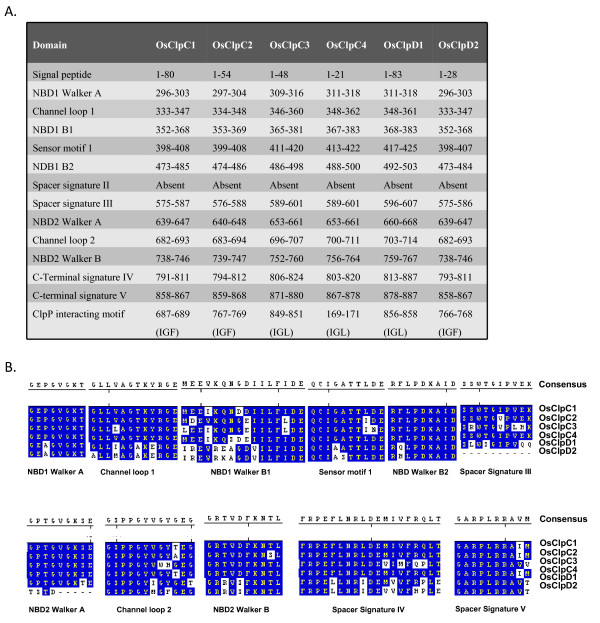
**A. Relative positions of domains in rice ClpC and ClpD proteins**. B. Consensus sequence of the different domains present in rice ClpC and ClpD proteins. Alignments were done using ClustalV of MegAlign module of DNASTAR and alignment pictures were modified for representation.

SMART and PFAM database were analyzed for retrieving information on various domains present in rice class I Clp ATPase proteins. In OsClpB-cyt, NBD1 and NBD2 span amino acids 209-398 and 607-710, respectively (Figure 2A). OsClpB-cyt also contains a spacer region which spans 494-553 amino acids; this spacer is characteristic of ClpB proteins. M-domain overlaps with spacer signature II. The signal peptides were predicted using Predotar and LOCTREE programs. Os02g08490 was predicted to be mitochondrial (hence shown as OsClpB-m), while Os03g31300 was predicted to be chloroplastic (hence shown as OsClpB-c). Although OsClpB-cyt is considered to be cytoplasmic, its amino acid sequence analyzed at LOCTREE program predicts that this protein is nuclear-localized and contains a putative nuclear localization signal (NLS) present from amino acid 476 to 483 (RKLKQREE). The architecture and the physical position of various domains in ClpC and ClpD proteins are shown in Figure 3. It is amply clear from this analysis that rice Clp C and D proteins belong to class I ATPases, containing two NBDs (Figure 3B). All the class I Clp ATPase proteins except OsClpC4 were seen to contain two copies of ClpN domain (Additional file 3). This domain present towards the N-termini is speculated to have a role in protein binding. ClpC2, C3, C4 and D2 proteins contained 35 amino acids long UVR domain between the two NBDs. UVR domains have been seen in proteins which have role in DNA recognition, repair and processing. The proteins containing UVR domain are reportedly able to interact with each other through this domain. OsClpC1 and OsClpC2 are predicted to be chloroplast-localized. On the other hand, ClpC3 is predicted to be mitochondrial/chloroplastic with equal probability and OsClpC4 is predicted to be present in plasma membrane. All the ClpC proteins identified to date in *Arabidopsis *and *Populus *are localized in the chloroplast where they interact with the chloroplastic protease ClpP.

Furthermore, 3-dimensional structures of OsClpBs were predicted using I-TASSER [58]. Of the various models suggested, structures which had a higher C-score were taken (C-score considered to be a confidence score for estimating the quality of predicted models by I-TASSER). Importantly, OsClpB-c, OsClpB-m and OsClpB-cyt showed almost similar structural arrangements as has been seen in case of *A. thaliana *ClpB (Additional file 4). The coiled-coil linker formed the central portion of the structures and the two NBDs flanked it. The predicted binding sites also showed the same pattern in the three ClpB isoforms (Additional file 5). From the structures proposed, it is suggested that OsClpB-c, OsClpB-m and OsClpB-cyt proteins are remarkably conserved with respect to their predicted protein binding sites.

In *E. coli*, complex formation between ClpA and ClpX with ClpP is mediated by a helix-loop-helix motif in which the tip of the loop contains an essential IGF/L tripeptide [59]. In case of rice Clp ATPase proteins, it was seen that except OsClpB-m, OsClpB-c and OsClpB-cyt (ClpB members), all other Clp class I ATPase proteins contain this tripeptide IGF/L motif (Figures 2,3). This motif was in general present towards the C-terminus except in case of OsClpC4 which had the tripeptide at its N-terminus.

### Expression of rice class I Clp ATPase genes

**Figure 4:**
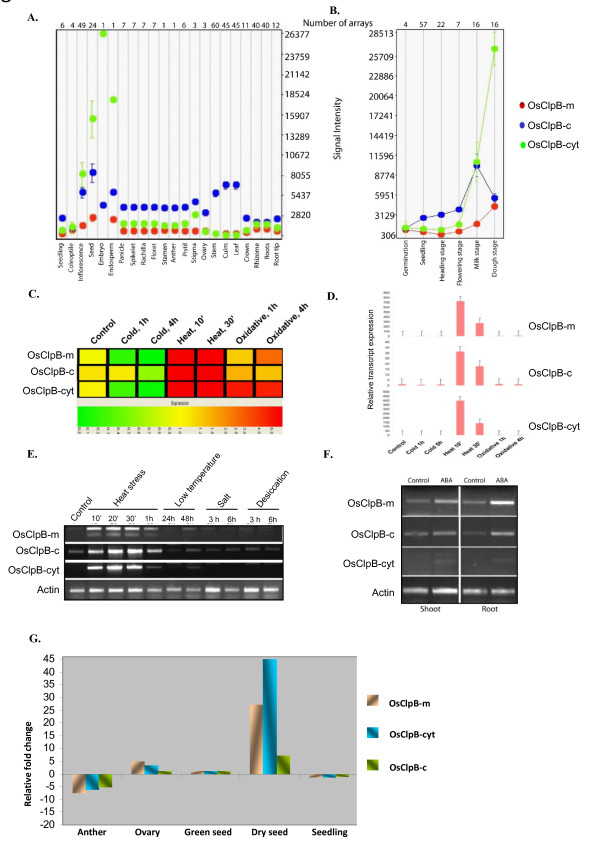
**Expression analysis of ClpB genes in various stages of rice plant based on the microarrays performed for rice**. **A**. Expression of *OsClpB *genes in different parts of rice plants. **B**. Expression of *OsClpB *genes during developmental stages. *In silico *analysis shown in **A **and **B **was performed using the Genevestigator database. The analysis included data from a total of 166 array experiments, and included 28 different tissues, organs or growth stages. **C**. Expression analysis of rice class I Clp ATPase transcripts in response to cold, heat and oxidative stresses. Hierarchical cluster display of expression profiles for *OsClpB *genes showing differential expression in rice (color bar represents the log2 expression values; green color representing low level expression, yellow shows medium level expression and red signifies the high level expression). **D**. Q-PCR analysis of the transcript expression of *ClpB-cyt, ClpB-c *and *ClpB-m *genes. The bars represent transcript level fold change values with respect to unstressed 10-d-old seedlings. Standard errors of the biological replicates are shown as error bars. **E**. Semi-quantitative RT-PCR based transcript expression analysis of rice *ClpB *genes under different stress conditions. The following stress conditions were used; HS: heat shock treatment (45°C; 10', 20', 30' and 1 h). LT: low temperature stress (8 ± 2°C; 24 h and 48 h). SS: NaCl stress (150 mM; 3 h and 6 h). DS: PEG treatment (12% PEG8000; 3 h and 6 h). PCR was performed for 25 cycles and products were resolved on 1% agarose gel. **F**. Semi-quantitative RT-PCR based transcript expression analysis of rice *ClpB *genes upon application of 100 μM ABA. PCR was performed for 25 cycles and products were resolved on 1% agarose gel.

**Figure 5:**
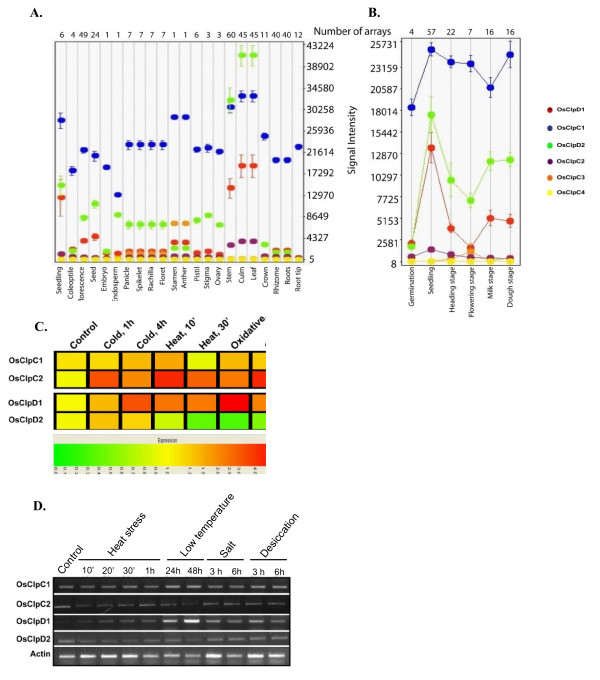
**Expression analysis of class I Clp ATPase genes in different parts of rice plant based on the microarrays performed for rice**. A. Expression pattern of *OsClpC *and *OsClpD *transcripts in different parts of rice. B. Expression pattern of *OsClpC *and *OsClpD *transcripts in different developmental stages of rice. *In silico *analysis shown in A and B was performed using the Genevestigator database. The analysis included data from a total of 166 array experiments, and included 28 different tissues, organs or growth stages. C. Expression analysis of rice Clp ATPase transcripts in response to cold, heat and oxidative stresses. Hierarchical cluster display of expression profile for rice *ClpC *and *ClpD *genes showing differential expression in rice (color bar represents the log2 expression values; green color representing low level expression, yellow shows medium level expression and red signifies the high level expression). D. Semi-quantitative RT-PCR of rice *ClpC *and *ClpD *genes under different stress conditions. The following stress conditions were used; HS: heat shock treatment (45°C; 10', 20', 30' and 1 h). LT: low temperature stress (8 ± 2°C; 24 h and 48 h). SS: NaCl stress (150 mM; 3 h and 6 h). DS: PEG treatment (12% PEG8000; 3 h and 6 h). PCR was performed for 25 cycles and products were resolved on 1% agarose gel.

Microarray data at Genevestigator database (https://www.genevestigator.ethz.ch/; [60]) were used for analyzing the transcript expression profiles of rice class I Clp ATPases in different tissues/developmental stages (Figures 4 and 5). In different developmental stages, the highest variation was found in expression of *OsClpB-cyt*: this gene showed minimal expression during germination, seedling, heading and flowering stages but showed high expression at milk and dough stages (Figure 4B). On the other hand, there was a gradual increase in the expression of *OsClpB-c *transcript from germination to milk stage and the expression declined during dough stage (Figure 4B). For expression of rice class I Clp ATPase genes in response to stresses, microarray profiling was carried out for analyzing expression during cold (5°C; 2 h and 4 h), heat (42°C; 10' and 30') and oxidative stress (10 mM H_2_O_2_; 1 h and 4 h) conditions. *ClpB *genes showed a notably enhanced expression during heat stress (Figure 4C). *OsClpB-cyt *showed enhanced expression during oxidative stress also although the magnitude of expression under oxidative stress was less as compared to heat stress. *OsClpB-c *and *OsClpB-m *showed almost identical response to the stresses (Figure 4C). Transcript expression for *ClpB *genes with Q-PCR matched with microarray data. While all three *ClpB *genes showed up-regulation under heat stress, the magnitude of the expression levels varied for the three genes (Figure 4D). Low levels of *OsClpB-m *and *OsClpB-c *transcripts were noted under unstressed conditions too. *OsClpB-cyt *transcript was strictly induced in response to heat stress (and to a lesser extent in response to oxidative stress; Figure 4D). The transcript abundance during heat shock was highest for *OsClpB-m *and lowest for *OsClpB-c *among the three *ClpB *ATPase genes. Semi-quantitative RT-PCR analysis showed distinct heat shock inducibility in all three ClpB genes (Figure 4E). *OsClpB-cyt *was mainly heat shock inducible. This transcript also showed a somewhat low expression in response to cold stress. All three *ClpB *transcripts were noted to be induced in response to exogenous ABA application to a comparable extent in shoot and root tissues of rice seedlings (Figure 4F). Expression of *OsClpB-cyt *was found to be much higher than other *ClpB *isoforms in dry seeds of rice, based on real-time PCR analysis (Figure 4G).

*OsClpC4 *appeared to be transcriptionally silent in microarray at Genevestigator. *OsClpC1 *showed maximum expression among the various ClpC genes. Levels of *OsClpC1 *transcript did not show much variation in expression; these transcripts were minimally expressed in endosperm. In general, there was almost similar expression profiles of *OsClpD1 *and *OsClpD2 *genes although there was variation in the magnitude of expression. *OsClpD2 *was seen to be expressed in anther and ovary but not in seed tissues (Figure 5A, B). Heat and oxidative stress inducibility was also relatively higher for *OsClpC2 *and *OsClpD1 *genes (Figure 5C). In the other Clp ATPases, only significant change marked was for low temperature inducibility of *OsClpD1 *(Figure 5D). Although microarray data showed that OsClpC2 was heat stress regulated, we did not find the same in semi-quantitative RT-PCR.

### Localization of OsClpB-c and OsClpB-m proteins

**Figure 6:**
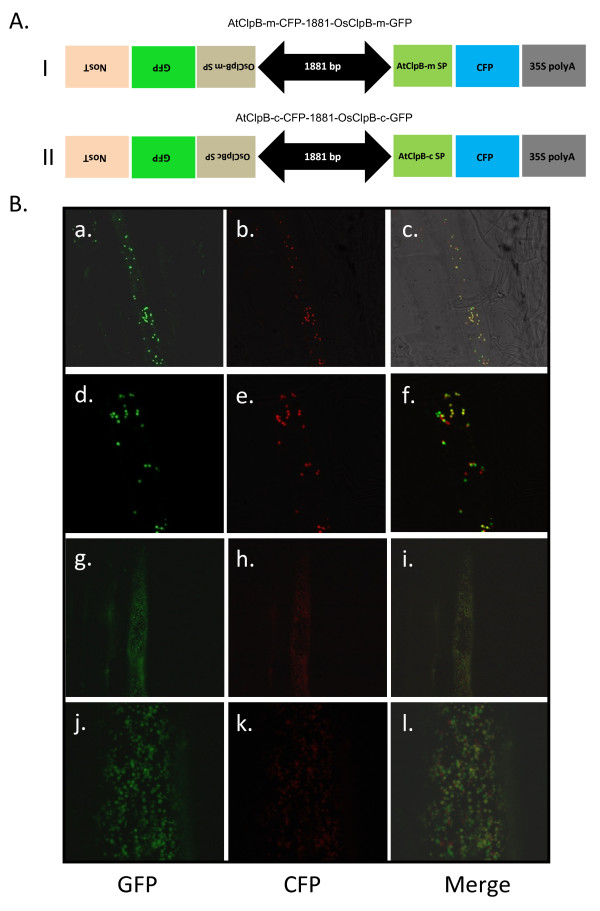
**A. Schematic representation of the constructs used for transient transformation of onion epidermal cells**. Panel I shows the AtClpB-m-CFP-1881-OsClpB-m-GFP construct while Panel II shows the AtClpB-c-CFP-1881-OsClpB-c-GFP construct. **B. Organellar localization of OsClpB-c and OsClpB-m proteins in transiently transformed onion epidermal cells**. **a, d**: OsClpB-c::GFP (**d **is enlarged view of a section of **a**); **b, e**: AtClpB-c::CFP (**e **is enlarged view of a section of **b**); **c, f**: OsClpB-c::GFP and AtClpB-c::CFP merged (**f **is enlarged view of a section of **c**). **g, j**: OsClpB-m::GFP (**j **is enlarged view of a section of **g**); **h, k**: AtClpB-m::CFP (**k **is enlarged view of a section of **h**); **i, l**: merged OsClpB-m::GFP and AtClpB-m::CFP (**l **is enlarged view of a section of **i**). CFP is shown in red for optimal image depiction.

*In silico *analyses shown in Table 1 predicted OsClpB-c and OsClpB-m proteins to be present in chloroplasts and mitochondria, respectively. To confirm their localizations, we designed two constructs namely AtClpB-m-CFP-1881-OsClpB-m-GFP and AtClpB-c-CFP-1881-OsClpB-c in this study. In AtClpB-m-CFP-1881-OsClpB-m-GFP construct, 90 amino acid long signal sequence (corresponding to 270 base pairs at the 5' end of ORF) from AtClpB-m were fused with CFP and used as a positive control and signal sequence from OsClpB-m in fusion with GFP expression as a test system; expression was driven by using a bidirectional promoter from a pair of rice protease inhibitor genes, for both the reporter proteins. Similar strategy was employed in construction of AtClpB-c-CFP-1881-OsClpB-c plasmid. In this case, 84 amino acids long signal peptide (corresponding to 252 base pairs at the 5' end of ORF) were fused to CFP and used as a positive control. Both plasmids were shot separately for transient transformation of onion epidermal cells. The fluorescence emitting from GFP and CFP proteins in both the constructs co-localized (Figure 6). This suggests that OsClpB-m is localized to the same sub-cellular compartment as does the AtClpB-m. Likewise, OsClpB-c is localized to the same sub-cellular compartment as does the AtClpB-c.

### Complementation of S. cerevisiae Δhsp104 mutant by rice class I Clp ATPase proteins

**Figure 7:**
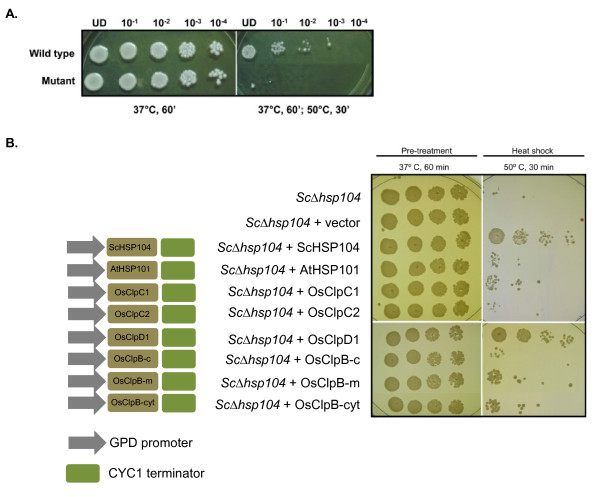
**A. Thermotolerance assay of *S. cerevisiae *(wild type) and *ScΔhsp104 *mutant cells**. B. Complementation analysis of yeast *Δhsp104 *mutant with various Clp ATPase genes. Left panel shows schematic representation of the different constructs used to transform *ScΔhsp104 *mutant cells and right panel shows thermotolerance assays with the transformed yeast cells.

Deletion of *hsp104 *gene in yeast (Sc*Δhsp104*) leads to thermosensitivity as the mutant cells are not able to survive heat shock (Figure 7A; [39]). Possible effect of different rice class I Clp ATPases in complementation of *Δhsp104 *mutant yeast cells was next analyzed. Different class I Clp ATPases were cloned in pGV8 vector under the control of GPD constitutive promoter to yield constructs pGV8-*OsClpB-cyt*, pGV8-*OsClpB-c*, pGV8-*OsClpB-m*, pGV8-*OsClpC1*, pGV8-*OsClpC2 *and pGV8-*OsClpD1 *(Figure 7B). Plasmid DNA containing different class I Clp ATPases were linearized and transformed into Sc*Δhsp104 *cells and selected on a medium lacking uracil to yield Sc*Δhsp104*-pGV8-*OsClpB-cyt*, Sc*Δhsp104*-pGV8-*OsClpB-c*, Sc*Δhsp104*-pGV8-*OsClpB-m*, Sc*Δhsp104*-pGV8-*OsClpC1*, Sc*Δhsp104*-pGV8-*OsClpC2 *and Sc*Δhsp104*-pGV8-*OsClpD1 *cell types. Sc*Δhsp104 *cells transformed with *AtHSP101 *(Sc*Δhsp104*-*AtHSP101*) and *ScHSP104 *(Sc*Δhsp104*-*ScHSP104*) were used as positive control while Sc*Δhsp104 *cells transformed with vector (Sc*Δhsp104*-vector) were used as negative control. Thermotolerance assay was carried out by giving a pre-treatment of 60 min at 37°C and then exposing cells to heat shock for 30 min at 50°C. Control and heat stressed cells were dotted on YPAD plates to a dilution of 10^-4 ^and kept at 28°C for 48 h and photographed (Figure 7B). We noted that positive control Sc*Δhsp104*-*ScHSP104 *grew better than Sc*Δhsp104 *and Sc*Δhsp104*-vector yeast cells. Positive effect was also seen in Sc*Δhsp104*-pGV8-*OsClpB-cyt*, Sc*Δhsp104*-pGV8-*OsClpB-m*, Sc*Δhsp104*-pGV8-*OsClpC1 *and Sc*Δhsp104*-pGV8-*OsClpD1 *cell types. Remarkably, Sc*Δhsp104*-pGV8-*OsClpD1 *cells grew even better than *ScΔhsp104-ScHSP104 *cells.

## Discussion and conclusions

We show in this study that rice genome has 9 entries (i.e. Os02g08490, Os02g32520, Os03g31300, Os04g32560, Os04g33210, Os11g16590, Os11g16770, Os12g12850 and Os05g44340) for the class I Clp ATPase protein family. In *Arabidopsis*, there are 6 class I Clp ATPase genes which specify 3 ClpB proteins (At1g74310-AtClpB-cyt; At5g15450-AtClpB-m; At2g25140-AtClpB-c), 2 ClpC proteins (C1, At5g50920; C2, At3g48870) and 1 ClpD protein (At5g51070) [17]. From the comparative details, it appears that 9 rice loci specify 3 ClpB (ClpB-cyt, Os05g44340; ClpB-m, Os02g08490; ClpB-c, Os03g31300), 4 ClpC (ClpC1, Os04g32560; ClpC2, Os12g12580; ClpC3, Os11g16590; ClpC4, Os11g16770) and 2 ClpD (ClpD1, Os02g32520; ClpD2, Os04g33210). Phylogenetic analysis using rice, *Populus *and *Arabidopsis *sequences showed that class I Clp ATPase sequences are significantly conserved. The tree showed three major clades, one each corresponding to ClpB, ClpC and ClpD proteins (Figure 1). ClpB proteins were further categorized into cytoplasmic, chloroplastic and mitochondrial isoforms based on TargetP, Predotar, LOCTREE and PSORT database. Cytoplasmic ClpB members included OsClpB-cyt, At1g74310 and Pt101.72; chloroplastic ClpB included OsClpB-c, At5g15450, Pt106.69 and Pt108.53 and mitochondrial ClpB included OsClpB-m, At2g25140 and Pt98.74. Although OsClpC3 and OsClpC4 are considered members of ClpC class, these proteins appear somewhat different from OsClpC1 and OsClpC2 proteins. All ClpC proteins identified to date are localized to the chloroplast where they interact with the chloroplastic protease ClpP. From the *in silico *analysis, it appears that ClpC3 on the other hand may be chloroplast/mitochondria localized while ClpC4 is membrane bound protein. Recently, AAA^+ ^ATPase chaperone CoxD protein in *Oligotropha carboxidovorans *is found to be present exclusively in cytoplasmic membrane [61]. A Clp ATPase in *Glycine max *has also been found to be membrane associated [62]. High temperature causes modifications in membrane functions, associated with alteration of membrane fluidity. In plant cells, membrane-based processes such as photosynthesis and respiration are especially important. Vigh et al [63] proposed that any alterations in the plasma membrane microdomains are well suited for sensing stress and re-tailoring the expression of various classes of HSPs. The chances of interaction of membrane-associated HSPs with the proteins involved in signaling increases as these molecules are also concentrated in the membrane rafts. ClpC4 protein of rice may have such an implication.

Importantly, *OsClpC2 *contained 691 nucleotides long intron in the 5'UTR and further harbored five upstream ORFs (uORFs) in the 5'UTR. The role of these uORFs in regulation, if any, needs to be worked out. Both *OsClpD1 *and *OsClpD2 *genes towards their 5' ends showed a **s**terile **a**lpha **m**otif (SAM) domain containing protein (Os02g32530 and Os04g33220): MSU rice gene model predicts that *OsClpD1 *and Os02g32530 gene pair and *OsClpD2 *and Os04g33220 gene pair may give rise to natural sense/antisense transcripts (NATs). Further analysis of *in vivo *regulation of ClpD proteins may reflect the significance of this observation. Domain analysis showed that OsClpB-cyt protein contains various motifs which are typical of HSP100 proteins. Spacer signature II motif seen in OsClpB proteins was notably absent from OsClpCs and OsClpDs (Figures 2, 3). Complex formation between ClpA and ClpX with ClpP is reportedly mediated by a helix-loop-helix motif in which the tip of the loop contains an essential IGF/L tripeptide (P-element; [59]). Weibezahn et al [64] engineered a ClpB variant termed BAP (ClpB-ClpP-loop; a variant protein in which ClpP interacting site of ClpA has been engineered into *E. coli *ClpB), which carried the P-element. Association of ClpP converted BAP from a refolding into a degrading disaggregase, demonstrating that this engineering brings in threading of aggregated proteins into the proteolytic chamber of ClpP. When rice class I Clp ATPase proteins were searched for the presence of IGF motif, it was seen that apart from OsClpB-m, OsClpB-c and OsClpB-cyt, all other proteins contained IGF/L tripeptide motif (Figures 2, 3). This observation may have an implication for differential function of ClpB as compared to other class I Clps. According to Kim et al [59], IGF/L tripeptide consensus is generally located between the sensor I and box VII motifs. However, OsClpC4 contained this motif towards the N-terminus. This may have some relation to the fact that unlike all other Clps, OsClpC4 is the only rice Clp that appears to be associated with the plasma membrane.

Cross-induction of *HSP *genes by diverse stresses is known [11,13]. Zou et al [65] have recently shown that the expression of nine different *OsHSP *genes was affected differentially by heat, cold, salt, desiccation as well as ABA. Hu et al [8] noted an extensive overlap of transcript levels in rice HSFs and HSPs in response to different stresses. Likewise, Mittal et al [9] noted cross-induction of HSF transcripts by diverse stresses in rice seedlings. On the same line, ClpATPase genes showed stress-related transcript expression. Agarwal et al [39] reported that *OsClpB-cyt*/*HSP100 *transcript in rice seedlings is induced strongly by heat and this transcript remains unaffected in response to salt stress, water stress, low temperature stress and ABA application. Hu et al [8] noted that *OsClpB-cyt *was regulated by heat shock as well as drought. In this study, microarray analysis showed that *OsClpB-cyt *transcript was up-regulated more than 5-folds both during heat stress as well as oxidative stress. *OsClpB-cyt *transcript was induced after low temperature stress (Figure 4E). This study is the first report for the cold induction of *OsClpB-cyt*, though at low levels. Wu et al [66] reported that rice *HSP100 *promoter responds to desiccation stress. In the experimental conditions used in the current study (PEG treatment), we failed to note induction of *OsClpB-cyt *transcript in response to desiccation stress. *OsClpB-c *was up-regulated during heat stress by up to 5-folds and during oxidative stress by up to 1.5 folds based on microarray analysis (Figure 4C). Q-PCR data showed that as with *OsClpB-cyt*, the relative abundance of *OsClpB-c *transcripts was far more responsive during heat stress as compared to oxidative stress. *OsClpB-m *gene, like its counterpart gene in *Arabidopsis*, was found to be heat and oxidative stress up-regulated (Figure 4). Q-PCR data showed that transcript abundance of *OsClpB-m *was highest during heat stress among the three *OsClpB *genes. On the other hand, AtClpB-m showed lowest transcript abundance among its different ClpB isoforms [17]. In a study on transcriptional profiling of genes responsive to hormonal treatments in rice, *HSP100 *transcript was found to be specifically induced in response to ABA [67]. We observe that transcriptionally, *OsClpB-m *and *OsClpB-c *respond more to ABA than *OsClpB-cyt *(Figure 4F).

*OsClpC1 *transcripts did not undergo any change under stress conditions while *OsClpC2 *was seen to be up-regulated (~ 3-4 folds) under heat, cold and oxidative stress conditions as seen in microarray experiment although we did not find the same upregulation in semi-quantitative RT-PCR analysis (Figure 5C). No amplicons were detected for *OsClpC3 *and *OsClpC4 *in the semi-quantitative RT-PCR analysis. This may indicate that these loci may in fact be transcriptionally silent under the conditions tested. However, *in silico *analysis of publicly-available microarray database shows *OsClpC3 *expression in stem, culm and leaf tissues (Figure 5A). Evidence for expression of *OsClpC4 *is yet awaited. Shen et al [68] reported that *OsClpD1 *is induced by water deficit and temperature stress in vegetative tissues. We observed that this gene was up-regulated by different stresses. In microarray data, *OsClpD2 *showed down-regulation during heat and oxidative stresses and a marginal up-regulation in cold stress (Figure 5C).

AtClpB-m and AtClpB-c have been shown to be localized to mitochondria and chloroplast, respectively [17]. We found that OsClpB-m is localized to the same location in the cell as AtClpB-m. Likewise, we noted that OsClpB-c is localized to the same location as AtClpB-c. We thus show that OsClpB-m and OsClpB-c are localized to mitochondria and chloroplast, respectively (Figure 6). It therefore, appears that these proteins may have specific functions in cellular organelles.

Yeast expressing HSP104 are typically 100- to 1000-fold more thermotolerant than yeast lacking HSP104, thus demonstrating the critical requirement for this protein in cell survival during extreme heat stress [69]. *Arabidopsis *ClpB-Cyt/HSP101 has been shown to overcome the temperature sensitivity of yeast *Δhsp104 *mutant [38]. Similar observations were noted for the ClpB-Cyt/HSP100 proteins from soybean [70] and rice [39]. This fact prompted us to analyze ability of diverse class I Clp genes in yeast mutant complementation assay. OsClpB-cyt showed reasonably good complementation ability. The capability of OsClpB-m appeared comparable to OsClpB-cyt in conferring tolerance. Our observation actually matches to an earlier experiment done with yeast [71]. This group expressed yeast Hsp78 protein which is mitochondrial isoform of the yeast cytoplasmic Hsp104 protein, in the cytoplasm of the Hsp104 mutant yeast cells. They noted that yeast Hsp78 protein complements the cytoplasmic Hsp104 yeast mutation. This experiment indicates that the organellar and the cytoplasmic ClpBs may have some degree of redundancy in their substrate recognition. Further, the target substrate proteins of different Clps are not yet defined. In due course when target proteins are established, it should be possible to address the question of mitochondrial ClpB complementing the cytoplasmic yeast *hsp104 *mutation more deeply. A significant observation in this assay test was the fact that OsClpD1 restores the mutation defect of the *ScΔhsp104 *yeast cells to a significant extent; Sc*Δhsp104*-pGV8-*OsClpD1 *showed growth comparable to that of the Sc*Δhsp104*-*ScHSP104 *yeast cells. ClpD expression in *Arabidopsis *has previously been seen to be associated during senescence [56] but there is no study that links expression of this gene to heat stress. The detailed mechanism behind this role of OsClpD1 remains to be worked out. It should be relevant to analyze how OsClpD1 expression alters transcript profiling in *Δhsp104 *mutant yeast cells. It should also be relevant to generate transgenic plants with ectopically higher ClpD1 protein and analyze their stress phenotype. Weibezahn et al [64] found that the development of thermotolerance in *E. coli *relies on the ClpB/KJE-mediated reactivation of aggregated proteins, whereas the removal of protein aggregates by degradation does not confer thermotolerance. Similarly in yeast, Tessarz et al [72] noted that the degradation of aggregated proteins results in loss of thermotolerance. They reported that the removal of aggregated proteins by HAP and ClpP is not sufficient for yeast cells to survive a severe heat shock, but survival demands on the reactivation of aggregated proteins. It can thus be inferred that disaggregation and reactivation of proteins is essential for cell survival after extreme heat stress, whereas the degradation of aggregated proteins is insufficient. It is possible that expression of OsClpD1 does this activity more efficiently than other class I Clps and hence its expression results in more thermotolerance than noted for other rice class I Clp proteins. Finally, it needs to be mentioned that although different Clp ATPases are noted to complement yeast *hsp104 *mutation in this study, it is possible that variations in expression levels of rice Clps in yeast which have not been determined, may also affect complementation efficiencies.

Finally, we wish to herein draw a comparable picture of *Arabidopsis *and rice class I Clp ATPases. There are some major differences in these two species for the number of ClpB, ClpCs and ClpDs nuclear genes. While *Arabidopsis *has 2 and 1 ClpC and ClpD members, respectively, rice appears to contain 4 and 2 ClpC and ClpD members, respectively. On the other hand, number of ClpB proteins appears same (3) in both rice and *Arabidopsis*. Both in rice and *Arabidopsis*, one each of ClpB type is present in cytoplasm, chloroplasts and mitochondria. It should be relevant to extend the above observations further to analyze what implications these might have in regulating heat response in these two contrasting plant species.

## Methods

### Growth conditions of rice and stress treatments

Rice [*Oryza sativa *L; cultivar Pusa Basmati (PB1), an indica type] seeds were washed with mild detergent and the detergent was removed by washing the seeds thoroughly with running tap water. Seeds were subsequently rinsed with 70% ethanol at room temperature for 45 s and washed with sterile distilled water 5-6 times to remove traces of ethanol. The seeds were soaked overnight at RT in dark before placing on cotton bed in a tray for germination. Seedlings were grown at 28 ± 2°C and 14 h light 10 h dark cycle maintained in growth room (light intensity ~ 250 μmol m^-2^s^-1^, humidity ~ 40%). For temperature stress, uniform-sized seedlings were transferred to beakers, which contained distilled water at 42 ± 1°C for heat stress (HS), at 5 ± 1°C for cold stress (CS), and 10 mM H_2_O_2 _at 28 ± 2°C for oxidative stress, and maintained at the requisite temperatures in BOD (for different time intervals as shown). For RT-PCR analysis, rice seedlings were subjected to heat, cold, salt and desiccation stresses. Salt stress was imposed by placing seedlings in beakers containing cotton pads soaked with NaCl solution, instead of distilled water. Roots and shoots were harvested and frozen in liquid N_2 _after desired durations of stress treatment. Desiccation stress was implied to rice seedlings by keeping in 12% PEG4000 as indicated. Subsequent to completion of the stress intervals, tissues were harvested, frozen in liquid nitrogen and kept at -80°C.

### Semi-quantitative reverse transcriptase PCR, Q-PCR and Microarray analysis

Total RNA was isolated from the control and stressed tissues from PB1 as per the standard protocol [73]. For semi-quantitative reverse transcriptase PCR, complementary DNAs were synthesized from 5 μg of total RNA primed with oligo (dT) primers using M-MLV reverse transcriptase (MBI Fermentas, Lithuania). RT-PCR amplification parameters were optimized to analyze individual target genes and gene specific primers were used. β-actin was amplified as an internal control. Q-PCR and microarray analysis were carried out as described earlier [9]. All primer details are provided in Additional file 6. Raw microarray data have been deposited in the Gene Expression Omnibus (GEO) database at the National Center for Biotechnology Information (NCBI) under the accession number GSE19983.

### Sequence analysis

ClpB homologs in rice were identified by performing BLAST search at the National Centre for Biotechnology Information http://www.ncbi.nlm.nih.gov/BLAST/ and MSU Rice Genome Annotation Project Database and Resource http://rice.plantbiology.msu.edu/ using the sequence of the ClpB-cyt/HSP100 (Os05g44340; Accession number AJ316025) as the query. The number and position of exons and introns in different genes were determined by comparison of the cDNAs with their corresponding genomic DNA sequences as well as by Spidey program http://www.ncbi.nlm.nih.gov/spidey/. In specific cases because of the lack of complete cDNA information, the ORFs were used for this analysis. The position of each gene on rice chromosomes was found by BLASTN search in genomic sequence of rice chromosome pseudomolecules available at MSU Rice Genome Annotation Project Database and Resource (Release 5). Multiple sequence alignments were done using the Clustal X (version 1.83) program [74] and the phylogenetic analysis was carried out by neighbor joining method [75]. The unrooted phylogenetic tree was displayed using the Treeview program [76]. The DNA and protein sequence analyses were performed using DNASTAR software.

### Cloning of signal peptides to confirm localization of OsClpB-m and OsClpB-c and transient transformation of onion epidermal cells

The sequences corresponding to signal peptides of OsClpB-c and OsClpB-m were amplified using AK069123 and AK287906 clones, respectively, as templates for PCR which were cloned upstream of GFP in pCAMBIA1302. Subsequently, signal peptide-GFP fusions along with NosT were amplified and cloned in pBCSK in BamHI and EcoRI sites and the plasmids were named as pBCSK-OsClpB-c-GFP and pBCSK-OsClpB-m-GFP. The signal peptides corresponding to *Arabidopsis *ClpB-c and ClpB-m were PCR amplified using *Arabidopsis *cDNA. The amplicons were cloned in the vector pAVA574 [77] to generate a fusion with CFP. Subsequently the signal peptide-CFP fusions were amplified along with the 35 S terminator and cloned in pBCSK-OsClpB-c-GFP or pBCSK-OsClpB-m-GFP vectors. The 1881 bp bidirectional promoter from a pair of rice protease inhibitor genes [78], was introduced in BamHI site. The plasmids so generated were named as AtClpB-m-CFP-1881-OsClpB-m-GFP and AtClpB-c-CFP-1881-OsClpB-c and were subsequently used for transient assays in onion epidermal cells as described previously [79] and analyzed for GFP and CFP expression using a Leica TCS SP5 inverted confocal microscope.

### Yeast transformation and thermotolerance assays of recombinant yeast cells

Yeast transformation was carried out as described previously [39,79]. For thermotolerance of yeast cells, a secondary culture of yeast cells (initial OD_600 _0.05) was started and incubated (25°C, 200 rpm) till OD_600 _reaches 0.15 (~ 3 h). OD_600 _of the secondary culture was also measured and if there was slight variation, it was normalized. Aliquots of 200 μl of the yeast cells from all the strains was made in MCTs which were placed immediately in a water bath set at 50°C for 30 min. For the constructs in *ScΔhsp104*cells, cells were given a heat pre-treatment at 37°C for 1 h. After stress, MCTs were immediately plunged into ice. For spotting, 10 fold serial dilutions (till 10^-4^) of the cells were prepared in sterile water and 5 μl of each was dotted on YPD-agar plates (YPD containing 1.8% w/v agar). The plates were dried in the laminar flow and incubated at 30°C for 2 d.

## Abbreviations

Clp: Casenolytic protease; HSP: heat shock protein; HS: heat stress; PCR: polymerase chain reaction.

## Authors' contributions

AS participated in the designing of the experiments and carried out the experiments. The computing work was done by AS. AS and US performed the semi-quantitative RT-PCRs and yeast thermotolerance assays. AS and DM performed the microarray and Q-PCR experiments. AG designed the experiments undertaken in this study. AG made contributions in the analysis of the data and interpretation of the findings. AS and AG together drafted the manuscript. All the authors read and approved the final manuscript.

## Supplementary Material

Additional file 1 **Properties of class I Clp ATPase proteins from *Populus trichocarpa *and *Arabidopsis***. The tables describe the properties of class I Clp ATPase proteins. Click here for file

Additional file 2 **Genomic organization of rice Clp ATPase genes**. Genomic organization of rice Clp ATPase genes based on the comparison of ORFs and genomic DNA. The exon-intron distribution is marked for the coding region of the genes and does not involve the UTRs. The scale above represents the nucleotides in kb. Black lines represent the introns while grey boxes represent the exons. The representation is to the scale. Click here for file

Additional file 3 **Domain architecture of rice ClpB proteins**. Schematic representation of various domains present in rice ClpB proteins. The proteins were aligned with respect to first NBD. SMART database was used to visualize the domains. Click here for file

Additional file 4 **Structural aspects of rice ClpB proteins**. **A**. Structure of *A. thaliana *ClpB monomer. **B**. Monomeric structures of OsClpB proteins as predicted at I-TASSER server. Structures with the highest C-score were chosen for all the proteins. **C**. Predicted binding sites present in the rice ClpB proteins. The predicted binding sites are shown in green spheres while N- and C-terminus in the model are marked by blue and red spheres, respectively. Click here for file

Additional file 5 **Details of the binding site predicted in OsClpB proteins using I-TASSER**. The binding sites were predicted in OsClpB proteins using I-TASSER. Click here for file

Additional file 6 **Primers used in the current study**. List of all the primers used in the analysis. Click here for file
