# Supplementary material for: Genome-wide analysis of rice ClpB/HSP100, ClpC and ClpD genes
Source: BMC Genomics. 2010 Feb 8;11:95. doi: 10.1186/1471-2164-11-95 (PMC2829514; doi:10.1186/1471-2164-11-95)
Supplement: Additional file 1 — Properties of class I Clp ATPase proteins from Populus trichocarpa and Arabidopsis. The tables describe the properties of class I Clp ATPase proteins. [file 1471-2164-11-95-S1.DOC]

Additional file 1. Properties of class I Clp ATPase proteins from *Populus trichocarpa* and *Arabidopsis.* aa- amino acids; pI- isoelectric point.

| **Proposed name** | **Type of Clp** | **Length (aa), pI** | **Predicted localization** | **Accession number** |
| --- | --- | --- | --- | --- |
| ***Populus trichocarpa*** | | | | |
| Pt101.7 | ClpB-cyt | 914, 5.96 | Cytoplasm | EEE77170 |
| Pt102.5 | ClpC1 | 924, 6.36 | Chloroplast | EEF06427 |
| Pt103.2 | ClpD1 | 939, 6.22 | Chloroplast | EEF05900 |
| Pt103.5 | ClpC2 | 932, 6.16 | Chloroplast | EEF06426 |
| Pt104.4 | ClpD2 | 948, 7.71 | Chloroplast | EEF96414 |
| Pt106.7 | ClpB-c1 | 949, 6.78 | Chloroplast | EEF04255 |
| Pt108.5 | ClpB-c2 | 967, 6.58 | Chloroplast | EEF85887 |
| Pt98.7 | ClpB-m | 877, 6.31 | Mitochondria | EEE92223 |
| ***Arabidopsis thaliana*** | | | | |
| At1g74310 (101.2) | ClpB-cyt | 911; 5.95 | Cytoplasm |  |
| At2g25140 (108.6) | ClpB-m | 964; 6.80 | Chloroplast |  |
| At3g48870 (105.7) | ClpC2 | 952; 6.25 | Mitochondria |  |
| At5g15450 (108.9) | ClpB-p | 968; 6.05 | Chloroplast |  |
| At5g50920 (103.4) | ClpC1 | 929; 6.59 | Chloroplast |  |
| At5g51070 (103.2) | ClpD | 945; 6.04 | Chloroplast |  |
