# Supplementary material for: Genome-wide analysis of rice ClpB/HSP100, ClpC and ClpD genes
Source: BMC Genomics. 2010 Feb 8;11:95. doi: 10.1186/1471-2164-11-95 (PMC2829514; doi:10.1186/1471-2164-11-95)
Supplement: Additional file 2 — Genomic organization of rice Clp ATPase genes. Genomic organization of rice Clp ATPase genes based on the comparison of ORFs and genomic DNA. The exon-intron distribution is marked for the coding region of the genes and does not involve the UTRs. The scale above represents the nucleotides in kb. Black lines represent the introns while grey boxes represent the exons. The representation is to the scale. [file 1471-2164-11-95-S2.PPT]

## Slide 1
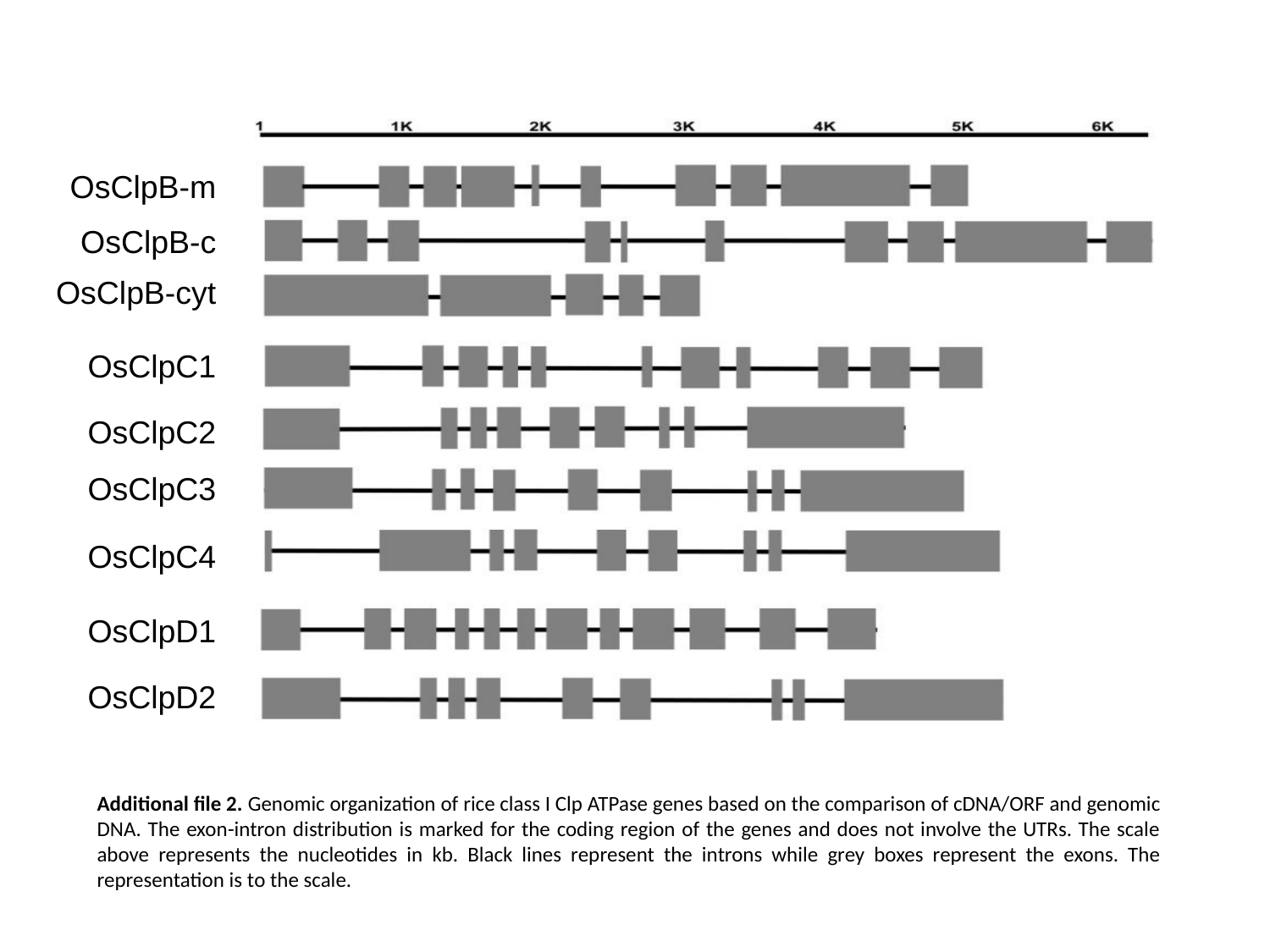

OsClpB-m
OsClpB-c
OsClpB-cyt
OsClpC1
OsClpC2
OsClpC3
OsClpC4
OsClpD1
OsClpD2
Additional file 2. Genomic organization of rice class I Clp ATPase genes based on the comparison of cDNA/ORF and genomic DNA. The exon-intron distribution is marked for the coding region of the genes and does not involve the UTRs. The scale above represents the nucleotides in kb. Black lines represent the introns while grey boxes represent the exons. The representation is to the scale.
