# Supplementary material for: Genome-wide analysis of rice ClpB/HSP100, ClpC and ClpD genes
Source: BMC Genomics. 2010 Feb 8;11:95. doi: 10.1186/1471-2164-11-95 (PMC2829514; doi:10.1186/1471-2164-11-95)
Supplement: Additional file 3 — Domain architecture of rice ClpB proteins. Schematic representation of various domains present in rice ClpB proteins. The proteins were aligned with respect to first NBD. SMART database was used to visualize the domains. [file 1471-2164-11-95-S3.PPT]

## Slide 1
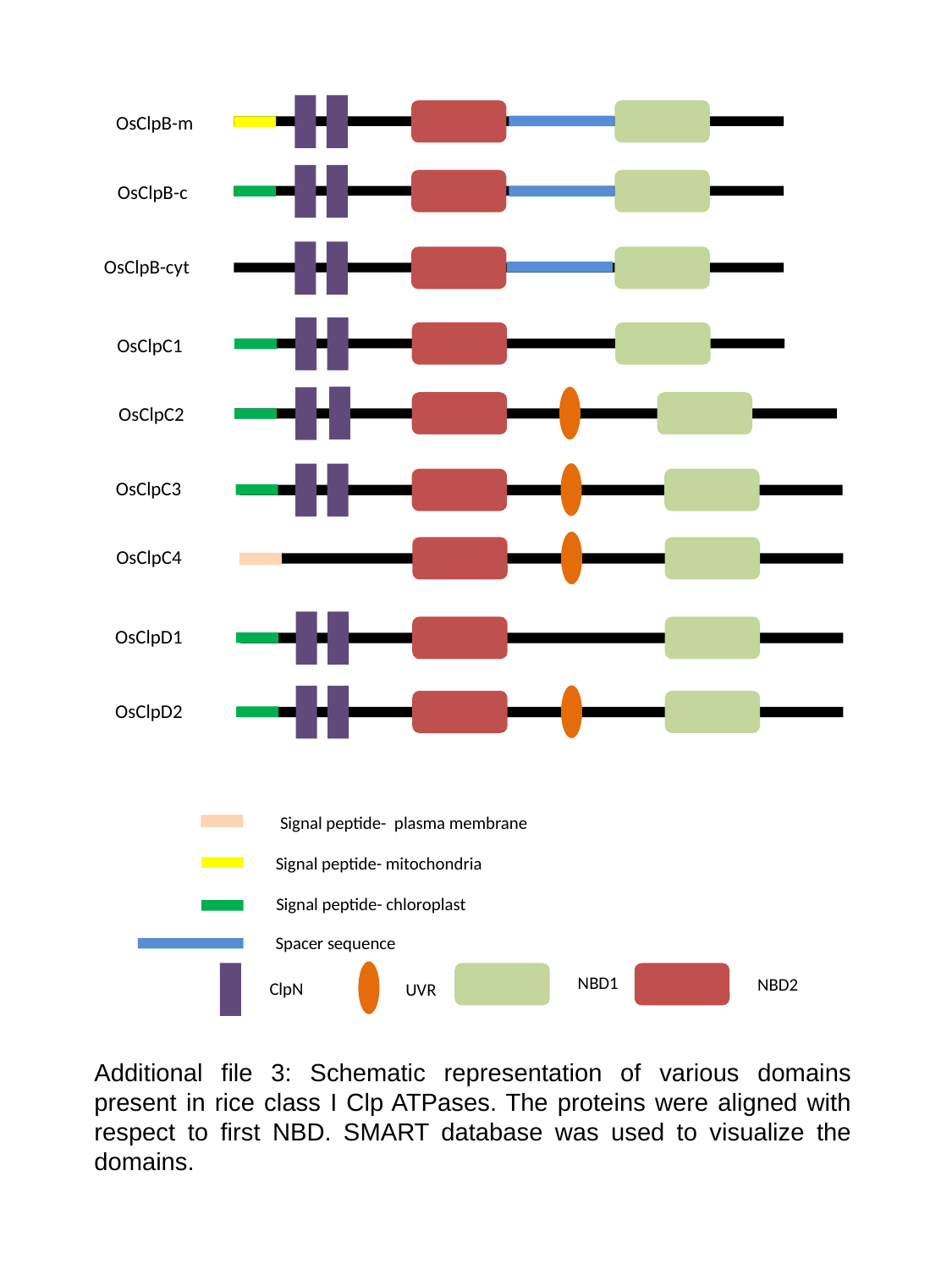

OsClpB-m
OsClpB-c
OsClpB-cyt
OsClpC1
OsClpC2
OsClpC3
OsClpC4
OsClpD1
OsClpD2
Signal peptide- plasma membrane
Signal peptide- mitochondria
Signal peptide- chloroplast
Spacer sequence
NBD1
NBD2
ClpN
UVR
Additional file 3: Schematic representation of various domains present in rice class I Clp ATPases. The proteins were aligned with respect to first NBD. SMART database was used to visualize the domains.
