# Supplementary material for: Genome-wide analysis of rice ClpB/HSP100, ClpC and ClpD genes
Source: BMC Genomics. 2010 Feb 8;11:95. doi: 10.1186/1471-2164-11-95 (PMC2829514; doi:10.1186/1471-2164-11-95)
Supplement: Additional file 4 — Structural aspects of rice ClpB proteins. A. Structure of A. thaliana ClpB monomer. B. Monomeric structures of OsClpB proteins as predicted at I-TASSER server. Structures with the highest C-score were chosen for all the proteins. C. Predicted binding sites present in the rice ClpB proteins. The predicted binding sites are shown in green spheres while N- and C-terminus in the model are marked by blue and red spheres, respectively. [file 1471-2164-11-95-S4.PPT]

## Slide 1
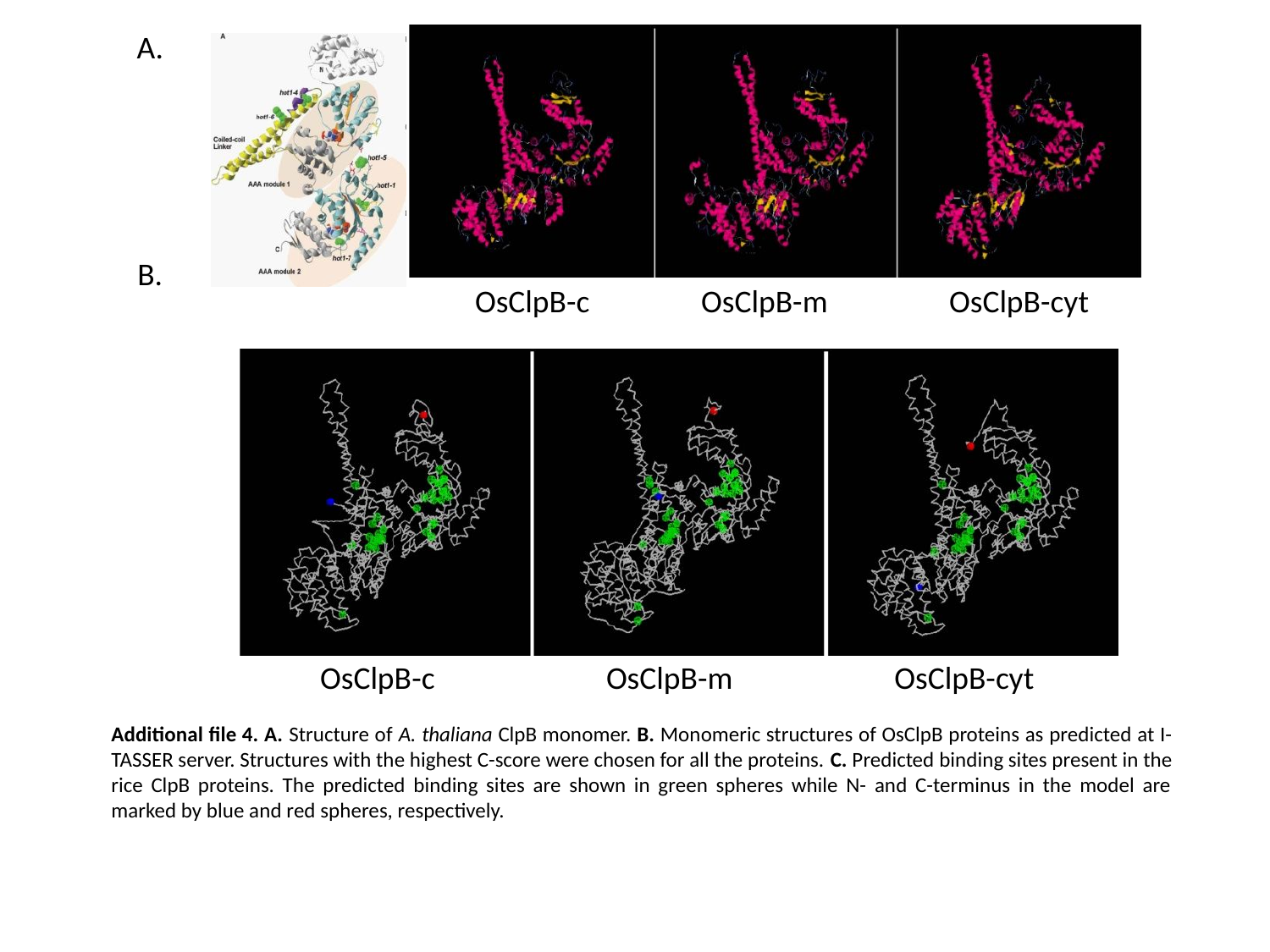

A.
B.
OsClpB-c
OsClpB-m
OsClpB-cyt
OsClpB-c
OsClpB-m
OsClpB-cyt
Additional file 4. A. Structure of A. thaliana ClpB monomer. B. Monomeric structures of OsClpB proteins as predicted at I-TASSER server. Structures with the highest C-score were chosen for all the proteins. C. Predicted binding sites present in the rice ClpB proteins. The predicted binding sites are shown in green spheres while N- and C-terminus in the model are marked by blue and red spheres, respectively.
