# Supplementary material for: Genome-wide analysis of rice ClpB/HSP100, ClpC and ClpD genes
Source: BMC Genomics. 2010 Feb 8;11:95. doi: 10.1186/1471-2164-11-95 (PMC2829514; doi:10.1186/1471-2164-11-95)
Supplement: Additional file 5 — Details of the binding site predicted in OsClpB proteins using I-TASSER. The binding sites were predicted in OsClpB proteins using I-TASSER. [file 1471-2164-11-95-S5.DOC]

Additional file 5. Details of the binding site predicted in OsClpB proteins using I-TASSER.

**OsClpB-m**

| SER | :118 |  | LYS | :167 |  | VAL | :334 |  | ILE | :335 |  | PRO | :362 |  |
| --- | --- | --- | --- | --- | --- | --- | --- | --- | --- | --- | --- | --- | --- | --- |
| GLY | :363 |  | VAL | :364 |  | GLY | :365 |  | LYS | :366 |  | THR | :367 |  |
| ALA | :368 |  | MET | :396 |  | ILE | :503 |  | LEU | :507 |  | PRO | :541 |  |
| ILE | :545 |  | ALA | :553 |  | LYS | :556 |  | MET | :557 |  | ARG | :727 |  |
| VAL | :728 |  | ILE | :729 |  | THR | :765 |  | GLY | :766 |  | VAL | :767 |  |
| GLY | :768 |  | LYS | :769 |  | THR | :770 |  | GLU | :771 |  | LYS | :774 |  |
| LEU | :787 |  | MET | :792 |  | SER | :793 |  | TYR | :795 |  | MET | :796 |  |
| ILE | :938 |  | ALA | :978 |  | ARG | :979 |  |  |  |  |  |  |  |

**OsClpB-c**

| SER | :107 |  | PRO | :262 |  | VAL | :263 |  | ILE | :264 |  | PRO | :291 |  |
| --- | --- | --- | --- | --- | --- | --- | --- | --- | --- | --- | --- | --- | --- | --- |
| GLY | :292 |  | VAL | :293 |  | GLY | :294 |  | LYS | :295 |  | THR | :296 |  |
| ALA | :297 |  | GLY | :326 |  | ILE | :432 |  | LEU | :436 |  | PRO | :470 |  |
| ILE | :474 |  | MET | :486 |  | ARG | :656 |  | VAL | :657 |  | VAL | :658 |  |
| THR | :694 |  | GLY | :695 |  | VAL | :696 |  | GLY | :697 |  | LYS | :698 |  |
| THR | :699 |  | GLU | :700 |  | LYS | :703 |  | VAL | :716 |  | MET | :721 |  |
| SER | :722 |  | TYR | :724 |  | MET | :725 |  | ILE | :866 |  | ALA | :906 |  |
| ARG | :907 |  |  |  |  |  |  |  |  |  |  |  |  |  |

**OsClpB-c**yt

| ALA | :24 |  | PRO | :182 |  | VAL | :183 |  | ILE | :184 |  | PRO | :211 |  |
| --- | --- | --- | --- | --- | --- | --- | --- | --- | --- | --- | --- | --- | --- | --- |
| GLY | :212 |  | VAL | :213 |  | GLY | :214 |  | LYS | :215 |  | THR | :216 |  |
| ALA | :217 |  | MET | :245 |  | ILE | :352 |  | LEU | :356 |  | PRO | :390 |  |
| ILE | :394 |  | VAL | :406 |  | ARG | :571 |  | VAL | :572 |  | VAL | :573 |  |
| THR | :609 |  | GLY | :610 |  | VAL | :611 |  | GLY | :612 |  | LYS | :613 |  |
| THR | :614 |  | GLU | :615 |  | LYS | :618 |  | LEU | :631 |  | MET | :636 |  |
| SER | :637 |  | TYR | :639 |  | MET | :640 |  | VAL | :777 |  | ALA | :817 |  |
| ARG | :818 |  |  |  |  |  |  |  |  |  |  |  |  |  |
