# Supplementary material for: Genome-wide analysis of rice ClpB/HSP100, ClpC and ClpD genes
Source: BMC Genomics. 2010 Feb 8;11:95. doi: 10.1186/1471-2164-11-95 (PMC2829514; doi:10.1186/1471-2164-11-95)
Supplement: Additional file 6 — Primers used in the current study. List of all the primers used in the analysis. [file 1471-2164-11-95-S6.DOC]

**Additional file 6: Primers used in the study**

**Primers used for semi-quantitative RT-PCR**

OsClpBC F 5’ CGCAGGGCAGGAAAGTGAGCTTCAC

OsClpBC R 5’ CCGCTCGAGTCAAACAGCCGGCAAAAATTTC

OsClpBCyt F 5’ ATTGTGATATTCGATCCCCTGTCCC

OsClpBCyt R 5’ GGAATTCCTACTCTTCGTCCATGCCG

OsClpBM F  5’ ATGACTTCAAACATTGGG

OsClpBM R 5’ GTTAGTCGCCAATGACTAG

OsClpC1 F 5’ GTTGGAAGCAGTGTCATTGAGAAGG

OsClpC1 R 5’ GCTCTAGATTAGACACTCAGAGCAGGTG

OsClpC2 F   5’ TAAGAATGAAGCTGTCCGTA

OsClpC2 R  5’ CATTCAGTACAATCACCTTC

OsClpC3 F 5’ ACCTGATGAAGCTAAAGAAC

OsClpC3 R 5’ GATCTCCTTCACCTCCAGCT

OsClpC4 F 5’ ATCAGCATACAGAACTTCAG

OsClpC4 R 5’ AATCCCCTTCTCTGACCTCT

OsClpD1 F 5’ ACAGAAGATACTGAATCAAG

OsClpD1 R 5’ GGAATTCTCAGAATGTCCGTGTCGG

OsClpD2 F 5’ CTGATACTGAAGAAAAGTCATATGC

OsClpD2 R 5’ GGAATTCTCAAAGTGTTGGTGTCGG

OsHSP74 F 5’ GGAATTCATGCCTCACCGGACAAGC

OsHSP74 R 5’ CCCAAGCTTCTATTCGTCGTCGTCAATCAC

Actin F 5’ CCCTATTGAGCATGGTATTG

Actin R 5’ CAGTTGTTGTAAAGGAATAA

**Primers used for Q-PCR**

OsClpBC F 5’ CTCGCCCGTCGAATCTTTT

OsClpBC R 5’ GCCGCTGGATGAACTTCTCT

OsClpBCyt F 5’ CGATCGAGCTGTGTGAATGAA

OsClpBCyt R 5’ AAAAAGGCATCATCGGAAAAAC

OsClpBM F 5’ CAGATAACTCCAGGAGAATTTACTGAGA

OsClpBM R 5’ CCACTACTTGTTGCTTCGACATTCTA

**Primers used for amplifying cDNAs for *hsp104* mutant yeast complementation**

AK058510 F  5’ GGAATTCATGGTTAAAGCAGAGACTG

AK058510 R 5’ CCGCTCGAGTTAGACACTCAGAGCAGG

AK066153 F 5’ GGAATTCATGTTTGAGAGGTTCACTG

AK066153 R 5’ CCGCTCGAGCTAACTATGCAGAAATATATC

AK068727 F 5’ GGAATTCATGGACATACCCTTC

AK068727 R 5’ ACGCGTCGACTCAGAATGTCCGTG

AK069123 F 5’ TCCCCCGGGATGGCCGCAGCGCC

AK069123 R 5’ CCGCTCGAGTCAAACAGCCGGCAAAAATTTC

AK069552 F 5’ ATGGAGAGAACCCTACTG

AK069552 R 5’ CTAGACAGTAAACG

OsClpB-M F 5’ TCCCCCGGGATGTCACGCGCCACCGCCGTG

OsClpB-M R 5’ AGGATATCCTAGTCATTGGCGACTAACTC

**Primers used in localization studies**

At2g25140 F 5’ GGATTCATGGCGCTAAGGAGATTATC

At2g25140 R 5’ CATGCCATGGGCTCCGTAAACTC

At5g15450 F 5’ GGAATTCATGGCGACGGCTACGACGACTG

At5g15450 R 5’ CATGCCATGGGTTCTGTGAAGCCTTCCTTGCTG

OsClpBC F 5’ CGGATTCATGGCCGCAGCGCCG

OsClpBC R 5’ TCAGATCTGACCTGACGGAGAGC

OsClpBM F 5’ CGGATTCATGTCACGCGCCACCG

OsClpBM R 5’ TCAGATCTGAGTACCTCGCCGCC

BamHI F (OCPI) 5’ CGGGATCCCTTCGCAAGGAAACAATCGAGAAG

BamHI R (OCPTI) 5’ CGGGATCCTCTGGTCACAGCAAATTAATG
